# Symptoms of Burnout Syndrome among Physicians during the Outbreak of COVID-19 Pandemic—A Systematic Literature Review

**DOI:** 10.3390/healthcare10060979

**Published:** 2022-05-25

**Authors:** Roxana Mihaela Claponea, Lavinia Maria Pop, Magdalena Iorga, Raluca Iurcov

**Affiliations:** 1 Faculty of Psychology and Education Sciences, “Alexandru Ioan Cuza” University of Iasi, 700554 Iasi, Romania; roxana.claponea@student.uaic.ro (R.M.C.); lavinia-maria.pop@umfiasi.ro (L.M.P.); 2 Behavioral Sciences Department, “Grigore T. Popa” University of Medicine and Pharmacy, 700115 Iasi, Romania; 3 Dentistry Department, Faculty of Medicine, University of Oradea, 410073 Oradea, Romania; riurcov@uoradea.ro

**Keywords:** physician, burnout, COVID-19, healthcare, pandemic, stress, medical profession

## Abstract

*Background*: Studies in the recent decades show that the medical profession has a high risk to develop burnout due to constant exposure to mental and physical suffering or death. The pandemic period induced additional stress for healthcare professionals due to the likelihood of a high rate of infection, long working shifts, using protective equipment, staying away from family, implementing new medical procedures. The present study is focusing on assessing the prevalence of burnout among physicians working in the healthcare system during the COVID-19 pandemic, and discovering the main factors associated with burnout syndrome among the population of physicians. *Material and methods*: A systematic review was conducted by searching PubMed, Wiley, and Google Scholar in November 2021. A total of 35 studies were eligible for the evaluation. *Results*: The samples ranged from 39 to 3071 physicians, and the overall burnout ranged from 14.7% to 90.4%. Sociodemographic characteristics associated with a high prevalence of burnout were the female gender, less experienced, not having children, and single marital status, associated with high levels of anxiety, depression, and stress in the female gender. The highest level of burnout among all the studies was 90.4% on a sample of physicians from the Republic of Korea, 80.2% among psychiatrists in Saudi Arabia, followed by a study in Ireland with a 77% level of burnout among senior and specialist physicians, and 74.7% prevalence of burnout for emergency physicians in USA. *Conclusions*: During the pandemic, the factors that contribute to burnout are the lack of personal protective equipment and the violence of issues related to organizational health; the high prevalence of burnout symptoms is associated with anxiety, depression, and stress.

## 1. Introduction

Burnout is defined as a prolonged response to emotional and interpersonal stressors and consists of three dimensions: emotional exhaustion, cynicism, and lack of professional effectiveness [1]. It is associated with absenteeism [2], poor performance, depression, psychosomatic problems, or drug use and most often affects employees in health, education, and justice systems [3]. Attention on burnout as a phenomenon began in the United States during the 1970s [1]. Corresponding to the original study, the dimension of emotional exhaustion has been defined as a condition of exhaustion characterized by the absence of tiredness, exhaustion, and fatigue. The dimension of cynicism was initially called depersonalization (taking into consideration the field of services), as well as unsuitable approaches toward clients, irritability, and lack of optimism. The dimension of professional efficiency was at first called “reduced personal achievement” and was characterized by low productivity, strain, and low self-esteem [4]. Burnout is often correlated with depression, as both are described by familiar components associated with sleep deficiencies, digestive disorders, feeling exhausted, decreased professional performance, reduced competence in communication, and the perception of desolation [5].

The medical profession was proved to be the most vulnerable to burnout because of the constant exposure to harsh situations such as mental and physical suffering or death. Therefore, healthcare workers, especially physicians, are at constant risk of exposing themselves to burnout, which could trigger attitudes that need immediate intervention, as well as raise awareness of the avoidance methods that could be used in order to prevent it [4,6,7]. The mental health of the professionals in this domain directly affects their ability to properly care for their patients. In recent years, there have been more and more initiatives aimed at maximizing well-being and minimizing the feeling of a high level of burnout among health professionals [4,5,6,7]. 

During the COVID-19 pandemic, medical personnel were exposed to high amounts of risk, worked long hours, suffered shortages of personal protective equipment, and had difficulty in treating patients in COVID-19 lockdown conditions. Moreover, the lack of relaxation activities, the exposure to high emotional demands, and uncertain security emerged into strain, fatigue, and burnout syndrome, which could generate medical errors. The pandemic had a huge impact on healthcare workers and challenged healthcare systems, generally, and the pandemic caused fear, anxiety, and depression on these workers [4,5,6,7,8].

The COVID-19 pandemic highlights the strengths and weaknesses of human resources in the field of health in terms of their competence and qualifications for such types of emergencies, as well as the link between public health issues and global health. The exceptional circumstances of COVID-19 have disrupted global health systems and forced sudden changes in the way individuals should be prepared for periods of uncertainty and change [9]. A pandemic is a disturbance that emphasizes stress and burnout due to the overwhelming work schedule. Nevertheless, gender issues (due also to gender-role inequalities) are becoming more and more acute, due to the long working hours spent at work by the frontline working force, such as the medical one, and the less time spent at home, which could also develop family issues and poor social relationships [6,7,8,10]. 

When societies and individuals face a sudden and radical change due to social isolation, there is a need for communication in the online environment, as well as teleworking. During the outbreak of pandemic, medical staff had to adapt their activity in order to provide medical care to patients. Online consultations, online training courses, and a rapid need to share medical information pressed doctors to use technological resources and to increase digitalization. While using technology to communicate with patients, physicians had to deal with social inequalities and ethical issues to maintain empathic communication in a virtual world and to rapidly develop their digitals skills [9]. One of the main causes of the burnout syndrome is the long-term exposure to stress. In the case of health professionals, it can lead to a decrease in the quality of care and, implicitly, to a decrease in patient satisfaction. It can also to an increase in medical errors and the risk of malpractice, staff fluctuations, alcohol and/or drug addiction, and even suicide. Therefore, the burnout experienced by medical staff is counterproductive for professionals and their families, as well as for patients and healthcare providers [11]. Fatigue at work takes into account both physical and mental elements and affects the general condition of employees. Moreover, fatigue can generate adverse health effects, such as chronic fatigue syndrome, burnout, and long-term musculoskeletal disorders [12]. The presence of certain requirements at work and the increased workload, personal conflicts, and lack of certain self-reliant methods of coping, social support, autonomy, and participation lead to the emergence of burnout that generates negative results, such as diseases, absenteeism, staff turnover, and reduced commitment [13]. 

Prior to the pandemic, medical healthcare personnel, represented by nurses and doctors, were characterized by requirements with an increased degree of difficulty, responsibility, and strong commitment [14]. The most common psychosocial risks for healthcare workers are workload, lack of organizational justice, emotional work, conflicting requirements, or role conflicts [15].

Since the start of the pandemic in 2020, 257,362,065 confirmed cases have been reported globally until November 2021, with 5,159,939 deaths caused by Sars-CoV-2 [16]. Many studies conducted during the first waves of the pandemic focused on identifying the levels of anxiety, depression, and stress among healthcare workers, along with the impact on their familial, professional, and social lives. However, after one year, many countries were still fighting with high levels of contagion and death, and doctors, especially those from the first line, were already exhausted. 

The aim of this study was to review the literature on the prevalence of burnout and its main factors, especially during the COVID-19 pandemic, summarizing and merging significant data to access a big picture of the coronavirus pandemic and the burnout effect on the physicians working in the healthcare system.

## 2. Materials and Methods

We conducted a systematic review of the scientific literature, following the Preferred Reporting Items for Systematic Review and Meta-analysis (PRISMA) guidelines [17].

### 2.1. Search Strategy

First, the literature in the field was investigated to discover if there existed systematic reviews on this specific issue regarding physician burnout during the COVID-19 pandemic. After the investigation, few studies were found, mainly analyzing healthcare professionals on the whole [18,19,20] or physicians of a specific speciality (pediatricians, psychiatrists, oncologists, etc.) [11,14].

Systematic research was conducted through the electronic databases Medline (PubMed), Wiley Online Library, and Google Scholar in November 2021. The design was assessed by using the Boolean operator “AND”, by using the terms “burnout” AND “physician” AND “COVID-19”. The suitable studies were selected by adopting a combined method that implied title and abstract reading, as well as a full-text evaluation. In addition, a well-performed review of references from articles, including previous systematic reviews [18,21], was conducted in order to prevent skipping articles by using digitized search.

### 2.2. Eligibility Criteria

1. The inclusion criteria followed the PICOS approach [22]:
2. The exclusion criteria used were (1) review articles and systematic review studies and (2) studies carried out before the COVID-19 pandemic.
3. Population (P): studies that included physicians.
4. Intervention (I): studies that analyzed the effects of MBIs and other scales on burnout in physicians.
5. Comparison (C): studies that presented the pre- and post-test results of the Maslach Burnout Inventory.
6. Outcomes (O): studies that measured the prevalence of burnout syndrome and factors associated with burnout.
7. Study design (S): cross-sectional, observational, prospective, mixed-methods, and multicenter longitudinal descriptive studies.

### 2.3. Study Selection and Data Extraction

After the initial search, eligible titles and abstracts were evaluated by two authors, along with the reading of full-text articles, which were individually revised for eligibility. All studies selected were added into Zotero, and after this step, duplicates were established and eliminated in a systematic process. 

An additional check was made by the method of checking the citations from the selected studies, which could also be considered a bias analysis. Next, full-text screening was completed in order to establish which studies were not relevant for the analysis, which fit the inclusion criteria, and which met the exclusion criteria.

Disagreements were resolved by consensus. The data extracted consisted of the following: study (authors, date, country, and design); population (sample size, sex, age, and clinical practice experience); burnout prevalence; main results of the studies; and burnout type of scale used.

## 3. Results

### 3.1. Study Selection 

**Figure 1:**
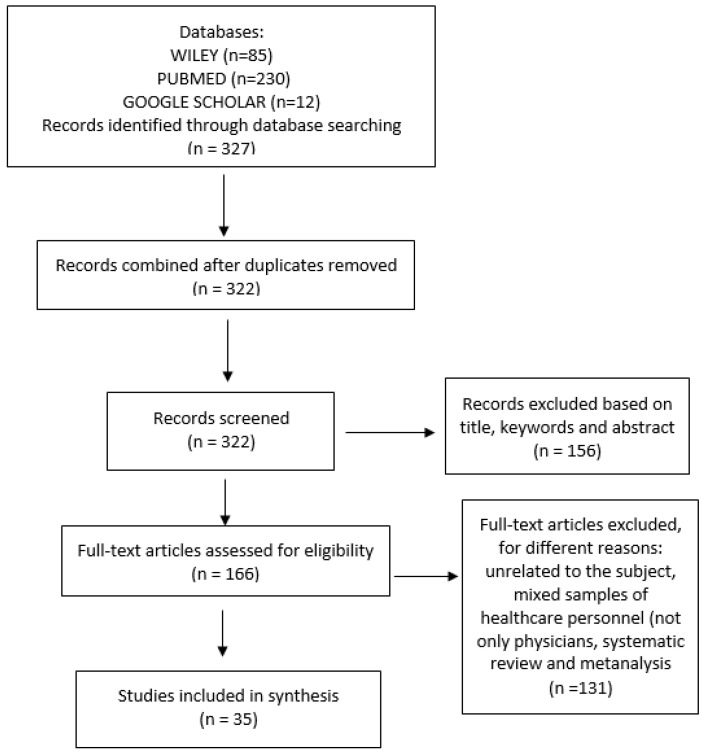
Flow Diagram.

A total of 315 articles were identified by checking PubMed and Wiley Library databases. In addition, by using Google Scholar, another 12 studies were identified. After following the initial screening and removing duplicates, 322 studies were left. Finally, a total of 131 studies were removed because they were found to be unrelated to the subject of interest, mixed samples of healthcare personnel (not only physicians), or systematic reviews and meta-analysis studies. For full-text evaluation, 166 studies were considered. A total of 131 studies were not taken into consideration, because they did not meet the inclusion criteria. A total of 35 studies were found to be eligible for the present research. The flow diagram of studies included in the research and the procedure of the selection can be identified in Figure 1.

### 3.2. Study Characteristics 

A total of 327 studies were identified. Following analyzing the title and abstract, as well as fully reading the text, 35 studies were included in our systematic review. The studies were conducted in 2020 and 2021, mainly in the USA (*n* = 13), followed by Brazil (*n* = 4), Turkey (*n* = 3), Argentina (*n* = 3), Canada (*n* = 2), Egypt (*n* = 2) Spain (*n* = 2), Jordan (*n* = 1), Saudi Arabia (*n* = 1), Portugal (*n* = 1), Ireland (*n* = 1), the Republic of Korea (*n* = 1), and Pakistan (*n* = 1).

All studies were conducted on healthcare professionals from a unique country, except for one study that targeted doctors from 20 countries and the USA.

Most of the studies are cross-sectional, observational, prospective non-comparative study design, and descriptive. One study was a multicenter longitudinal descriptive study, and the physicians who were included in the research responded to the three surveys, with only 40 respondents included in the research. The number of doctors involved in the studies varied from 39 to 3071.

The articles included in the present review of the literature gathered data from mixed medical specialities (so they declared that the questionnaires were distributed to physicians in general) but also studies focused on specific specialities, such as cardiology, emergency physicians, physiatrists, oncologists, pediatricians, neurosurgeons, orthopedic surgeons, internists, and family doctors.

Detailed information about each study is reflected in Supplementary Material File S1. 

### 3.3. Burnout Instruments and Other Tools

For measuring burnout, one of the most applied instruments is the *Maslach Burnout Inventory* (MBI), which is estimated to be used in 88% of all burnout publications. MBI contains three dimensions, namely emotional exhaustion, depersonalization, and reduced personal accomplishment. The three dimensions mentioned are measured by using 22 items on a seven-point Likert scale, starting from zero, which is never, to six, which means every day. If a high score is registered for emotional exhaustion and depersonalization, and a low score for the dimension of personal accomplishment, burnout is pointed out with the intensity of low, medium, and high [1]. In our literature review, 60% of the studies (*n* = 21) used this psychological instrument.

The other tools used to identify the level of burnout were as follows:

The 10-item Burnout Measure—Short Version (BMS), the short version of the burnout measure (bm) [23], includes 21 items measured on a seven-point Likert scale, evaluating individual’s physical, emotional, and mental exhaustion [24] (two studies). 

The Mini-Z Burnout Assessment consists of measuring satisfaction, stress, burnout, and the risk factors associated with them, and it is validated against the Maslach Burnout Inventory [25] (two studies). 

The Copenhagen Burnout Inventory (CBI), consists of three scales assessing personal burnout, work-related burnout, and client-related burnout (one study) [26].

The Stanford Professional Fulfillment Index (PFI) consists of 16 items evaluating workload exhaustion, depersonalization, and professional fulfillment [27] (two studies).

The Maslach Burnout Inventory Human Services Survey (MBI-HSS) consists of 22 questions which are categorized as emotional exhaustion, depersonalization, and personal accomplishment measured on a seven-point Likert scale (seven studies) [28].

The Oldenburg Burnout Inventory (OLBI) instrument is measured as absent or mild burnout or moderate or severe burnout (one study) [29]. 

The six COVID-19 burnout emotions measure related burnout symptoms based on emotions related to doctors’ burnout, such as emotional exhaustion, depression, helplessness, and anxiety (one study) [30].

Some other psychological tools were used in order to identify the factors that are related to the presence of burnout and which variables are the most important. Many studies focused on identifying burnout in relationship with COVID 19 anxiety, fear of infection, compassion fatigue, depression, and quality of life.

The Compassion Fatigue and Satisfaction Self-Test (CFST) contains 54 statements, with 18, 13, and 23 items on the compassion fatigue, burnout, and compassion satisfaction [31].

The Fear of COVID-19 scale (FCV-19S) is a self-report measure aimed at assessing fear of COVID-19 and was also found to correlate with anxiety and depression. The scale consists of seven items measured on a five-point Likert scale pertaining to emotional fear reactions toward the pandemic [32]. 

The Hospital Anxiety and Depression Scale (HADS) consists of 14 items; it was developed by Zigmond and Snaith [33] and determines anxiety and depression [34].

Moreover, the World Health Organization Quality of Life Scale (WHOQOL-BREF) was used for some research to assess quality of life [35].

### 3.4. Prevalence of Burnout Syndrome

Various levels of burnout syndrome were identified in the studies selected, based on country, sample size, or physician speciality. The highest level of burnout among all the studies was 90.4% on a sample of physicians from the Republic of Korea [36], followed by a study with 80.20% in a study carried out in Saudi Arabia on a sample of psychiatrists [37], followed by a study in Ireland, with 77% level of burnout for senior specialist physicians [38], and another in the USA, with a 74.70% prevalence of burnout for emergency physicians [39]. 

The MBI scale was used in most of the studies taken into consideration for the analysis. The lowest scores of burnout were established in a study with a sample of 163 physicians in the USA [40], with an overall score of burnout of 14.70%, followed by another one on a sample of 225 residents in the USA with a 19.60% prevalence of burnout [41]. 

A deep analysis for the three dimensions of burnout revealed that emotional exhaustion had a high prevalence in a sample of 302 physicians in Canada [42] and was significantly higher in physicians exposed to violence [43]. Depersonalization prevalence could be established in a sample of 101 psychiatrists in Saudi Arabia [37] and reduced personal accomplishment in a sample of 1015 internists from Spain [44].

### 3.5. Sociodemographic-, Familial-, and Financial-Related Factors

Sociodemographic factors such as *being younger* [39,41,45,46,47], *female gender* [37,40,41,45,48,49,50], *unmarried status* [28,51], *not having children* [46,47], and *junior experience* [37,49] were correlated with high levels of burnout. 

Furthermore, *emotional exhaustion* was associated with female gender [42,45], *less professional experience and unmarried status* [28], and *being younger* [52].

*Depersonalization* was correlated with *male gender* [45] *residents and those who worked in COVID-19 units* [28]. *Personal accomplishment* was negatively associated with *professional experience* [28], *infection or death from COVID-19 among colleagues or relatives* [45], and *being a female* [42]. *Female gender* was also associated with a *poor work–life balance* [41].

In the study conducted by Guercovich et al. [53], 72.3% of the included doctors answered that the family’s income was reduced during the COVID-19 pandemic, and, at the same time, the financial or psychological support from the institutions was missing. More than half of the doctors (67%) included in the study conducted by Jha et al. [54] in the USA responded that in-house billing was responsible for their increased level of burnout. A monthly income of less than 900 € predicted lower levels of job satisfaction, leading to higher burnout scores [48]. Moreover, the financial worries and the fact that the money earned during the pandemic would not be enough to support the needs of the family, sometimes even overcame the fear of death [55].

### 3.6. Institutional and Occupational Factors

Physicians exposed to COVID-19 patients were more likely to feel high levels of anxiety [56]. 

Burnout levels were higher for doctors who needed to buy personal equipment by themselves [45,48]. 

Working night shifts and working long hours were also correlated with burnout [48,56]. Emotional exhaustion was higher for those who worked in COVID-19 units and for those infected with COVID-19 [57], and mental well-being was associated significantly with COVID-19 infection and personal accomplishment [28]. 

Physicians working in the emergency department and residents reported a higher frequency of burnout syndrome [56]. The frequency of burnout syndrome, anxiety, and depression was significantly higher among residents and physicians working in the emergency departments [56]. 

Mental well-being was significantly negatively associated with COVID-19 infection and positively associated with personal accomplishment [28]. Depersonalization was reported more frequently in those who worked in COVID-19 units than in those infected with COVID-19. Personal accomplishment was observed to be lower in those with a connection to COVID-19 patients [28]. The management of COVID-19 patients was correlated with burnout [44]. Being an active physician in the fight against COVID-19 was negatively correlated with the presence of burnout [52]. Oncologists were more likely to feel low levels of personal accomplishment [53].

### 3.7. Mental Health and Quality of Life 

Emotional exhaustion and depersonalization were greater in those physicians with lower mental well-being scores, with personal accomplishment directly proportional to mental well-being [28]. 

Choosing the career unwillingly was also positively associated with burnout [52]. Trainees taking care of COVID-19 patients were more likely to experience burnout [58]. In-house billing and electronic medical records were reported as stressor factors for physicians in the USA [54]. 

Trainees with greater concern over missed educational opportunities were more likely to be experiencing burnout [59], and a great number of physicians reported changing their behavior toward family and friends, especially by decreasing signs of affection [60].

Symptoms of moderate-to-severe depression and anxiety have a negative impact on doctors’ quality of life, so anxious physicians have had a lower quality of life compared to the non-anxiety group [61]. There were also statistically significant differences between the average scores of the four areas: physicians’ quality of life and inadequate training related to the pandemic, physicians’ dissatisfaction with the measures of personal protective equipment in the hospital, and history of COVID-19 medical condition [62]. In addition, smoking and drinking habits may be associated with poor health-related quality of life and poor mental health, with alcohol intake and stimulant use being higher in the anxiety group during the COVID- 19 pandemic [61].

### 3.8. Depression, Anxiety, Stress, and Sleep Disorders

Burnout was positively associated with a history of depression [41] or anxiety [40]. Females were more exposed to depression, anxiety, and stress [36,63]. 

Another issue concerning burnout is related to the lack of sleep, mainly for elderly physicians [64]. Additionally, sleep disturbance was significantly associated with the physician’s age [61].

Anxiety and COVID-19-related burnout were significantly associated with the physician’s age, gender, and years in practice.

Some studies identified an increase rate of consumption of pills (for stress and sleep-related problems) and an increased rate of alcohol consumption among doctors with high levels of burnout [44,60,61].

### 3.9. Professional Related Factors

The higher levels of burnout were identified among participants from various categories:

1. Medical speciality—infectious diseases physicians [36], psychiatrists [37], and emergency physicians [39];
2. Category of physicians—residents [28,29,43,56].
3. University teachers—PA was low among research assistants and in participants working in university research hospitals [34].
4. Education and professional opportunities—High levels of burnout were identified in a study conducted among physicians in the USA [59]. Trainees in pathology or radiology had greater odds of concern for missed educational opportunities as compared to trainees in medicine.

## 4. Discussion

Burnout syndrome in the medical profession is a significant public health problem, as it has negative effects on the well-being of the doctor, patient care, and the health system [65]. Doctors who are unaware of their condition, ignore signs of exhaustion, and continue to work are more likely to have low labor productivity, exhaustion, and poor quality of care [66,67]. In addition, there is an increased economic burden of training and recruiting new staff when experienced physicians are exposed to burnout [68].

The level of burnout in the COVID-19 pandemic from the analysis studies is different between regions and physician specialities. We can also identify similar results for the studies related to burnout prevalence [28,29,39,44,49,58], where emotional exhaustion or depersonalization was observed in more than 50% of the participants. 

Similar to other studies, the prevalence of burnout was reported by more than 50% of the respondents from the healthcare system, with more than 50% experiencing emotional exhaustion and depersonalization and low personal accomplishment [18]. The level of physician burnout during outbreak diseases (including COVID-19) was reported to range from 15% to 75%. Those numbers were registered despite the country or the type of disease experienced (e.g., Ebola or A virus).

This review found several variables associated with burnout. Considering the socio-demographic elements (younger age, female gender, unmarried marital status, and less experienced doctors), we can say that all of these factors have a particularly important role in the burnout syndrome, with doctors being more vulnerable to psychological symptoms, including burnout, depression, and anxiety [21]. 

In the context of the pandemic, the volume of work [48,56], the lack of resources (for example, personal protective equipment) [45,48], and the perceived threat of COVID-19 [56,57] had an impact on the health of medical staff, both in terms of exhaustion and other mental problems. Such results are similar to the ones found in our systematic review, emphasizing the huge impact of a pandemic on the healthcare system, specifically on physicians. 

The studies showed that doctors who are supported or feel supported by their family or loved ones experience less burnout compared to those who do not. The study by Khalafallah et al. [47] found that both having children and spending more time with family members during the pandemic were independently associated with a lower risk of burnout. Asghar et al. [28] agreed that having a partner or being married was associated with a low risk of burnout, with emotional exhaustion being lower in physicians who were married (*p* = 0.026). 

Personal accomplishment was negatively associated with the following variables: professional experience, infection or death from COVID-19 among colleagues or relatives, and being a female. The results identified by the present review of the literature are strongly supported by the other studies that focused on fear of infection with SARS-Cov-2 identified during the first wave of the pandemic, when burnout levels were not measured yet. For example, Iorga et al. [11] showed that, during the outbreak of the pandemic, more than 10% of ob-gyn doctors used pills to cope with work stress, and 25% of them had sleep disorders, along with appetite loss. The authors also identified that working under the stress of an infection with SARS-Cov-2 causes a lot of pressure and determines changes in personal, familial, social, and professional life. Another medical speciality extremely touched by the pandemic was dentistry, with a high risk of infection. A quarter of the dentists included in the research by Iurcov et al. [69] declared that they had periods when they lived away from home, being afraid of transmitting the disease to their family members; this especially applied to doctors with children. Dentists with chronic diseases showed a higher level of anxiety when following the news and stories related to COVID-19 on TV, the media, or social networks.

It is noteworthy that various studies that have focused on identifying the presence of burnout in physicians during the pandemic have used MBI or short forms of this tool. Along with these psychological tools, other scales were applied to the subjects, so many different measures of symptoms of burnout syndrome were used in the reviewed papers. It is also important to mention that some authors identified that studies that used the MBI test identified the effect between burnout and anxiety as being lower compared to the ones that used a different burnout measure [70,71]. The same results were pointed by some other researchers that reported a lower association between burnout and depression compared to the studies that used other burnout measures, where the association between burnout and depression was higher [72,73].

Strengths and limitations: This review had several limitations that need to be acknowledged, including the small number of papers contained in it, methodological discrepancies, and the heterogeneity of reporting approaches. In addition, there is no comprehensive tool to measure events that take place outside the workplace, and since other major disorders are often overlooked, or burnout assessments do not consider the fact that such triggers may overlap with other mental disorders (such as depression, anxiety, and PTSD), it is difficult to understand exactly how plausible the burnout data are. Moreover, this review did not define how physician burnout varies among practitioners from different specialties of medicine. It should also be noted that only one study is longitudinal; therefore, it is difficult to identify that there is a significant difference between the levels of burnout before and during the pandemic, so the results of the studies should be viewed from this perspective. Another limitation of the study is represented by the cultural factors that could be related to the results of the research considered for this review, factors that were not included in the studies and could bring important explanations for some of the results. However, this review brings more knowledge to the universal phenomenon of physician exhaustion that has a significant impact on patient care, and the facts presented in this review should be considered in assessing and resolving this global issue. 

### Reflections and Planning

Studies published during the first waves of the pandemic did not accurately highlight the fine changes in terms of burnout among physicians. Each country has a specific medical system, and the institutional, political, social, and cultural approaches are diverse. That is why the studies mentioned in this literature review have brought a different perspective on how doctors coped with the pandemic in its first waves. 

The results of studies in various countries are important, even if they are achieved very shortly after the onset of the pandemic and the imposition of restrictions, and, at the same time, they show how various medical specializations have resonated with changes in medical systems. The results are important both to explain the impact on physicians and to identify, in the long term, new strategies that can be applied by health policymakers. The results of the present review are providing another perspective on burnout among doctors. Previous research on healthcare specialists before the pandemic showed an increased level of burnout [74,75], and a literature review conducted on healthcare specialists [76] identified that the burnout levels increased during the pandemic. Thus, the present paper adds new perspective about frontline and non-frontline physicians. 

## 5. Conclusions

The present paper provided an overview of the prevalence of burnout among physicians during the outbreak of the COVID-19 pandemic. The research put forward a high prevalence of burnout symptoms associated with other personality traits, such as anxiety, depression, and stress. There are several factors contributing to burnout, in regard strictly to the pandemic conditions, such as the lack of personal protective equipment and the violence of organizational healthcare aspects. Further research is needed in order to perform a better overview of the pandemic. It is necessary to perform a meta-analysis to show the intervention methods that could be implemented to effectively prevent or reduce burnout levels.

## Data Availability

Not applicable.

## Supplementary Materials

The following supporting information can be downloaded at https://www.mdpi.com/article/10.3390/healthcare10060979/s1. File S1: Study characteristics.
