# Supplementary material for: Symptoms of Burnout Syndrome among Physicians during the Outbreak of COVID-19 Pandemic—A Systematic Literature Review
Source: Healthcare (Basel). 2022 May 25;10(6):979. doi: 10.3390/healthcare10060979 (PMC9223230; doi:10.3390/healthcare10060979)
Supplement: Supplementary file 1 [file healthcare-10-00979-s001.zip › healthcare-1691568-File S1.pdf]

Table S1: The articles included in the review.

Author, Year	Country	Study design	Sample	Burnout prevalence	Main results	Burnout Scale
Abdelhafiz et al, 2020	Egypt	Cross-sectional study	220 physicians	Burnout frequency among the research group was 36.36%.	Burnout development was raised two times with the need to buy personal protective equipment and was less likely to develop in doctors with older age. Male gender was a predictor of Depersonalization (D), and female gender was significantly related to higher Emotional Exhaustion (EE) - was correlated with infection or death from COVID-19 among colleagues or relatives. Professional Achievement (PA) was low because of infection or death from COVID-19 among colleagues or relatives.	Maslach Burnout Inventory MBI-HSS
Abdelghani et al, 2020	Egypt	Cross-sectional study	320 physicians	For burnout symptoms, high EE, high D, and low PA were reported by 20, 71, and 39% of physicians, respectively.	COVID-19 virus infection was positively associated with anxiety, depression, EE, and D symptoms, and negatively correlated with PA and all quality-of-life domains.	MBI
Al-Humadi et al, 2021	U.S.A.	Cross-sectional study	225 residents/ fellow physicians	19.6% prevalence of burnout	Female physicians registered less work-life balance and a high level of burnout, which was negatively associated with age and positively associated with a history of depression or anxiety and the number of times on call	MBI
Alrawashdeh et al, 2021	Jordan	Cross-sectional study	973 physicians	The prevalence of burnout was 57.7%	Female gender, working at highly loaded hospitals, working for long hours, doing night shifts, insufficient access to personal protective equipment, and being positively tested for SARS-CoV-2 were positively associated with burnout.	BMS – short version
Alwashmi & Alkhamees, 2021	Saudi Arabia	Cross-sectional study	101 physiatrists	80.2% of the participants experienced burnout which was correlated with scoring high on the EE and/or the D.	Physiatrists who work in tertiary centres are more likely to experience burnout. Physiatrists in-training and with less experience are more likely to experience burnout. Female physiatrists are more likely to experience burnout. Burnout was statistically not significant in relation to stress, anxiety, or depression.	MBI-HSS
Appiani et al, 2021	Argentina	Cross-sectional study	302 physicians	The prevalence of burnout syndrome was 73.5 %	Physicians who had a positive score were significantly younger. Burnout was associated with having less seniority, being a resident, carrying for patients with potential or confirmed COVID-19 infection, having COVID-19-like symptoms, and working 24-hour shifts. Burnout, anxiety, and depression were significantly higher among residents and physicians working in the emergency department.	MBI

Asghar et al, 2021	Pakistan	Cross-sectional study	87 physicians	EE was observed in 54% of participants. D was observed in 77%. Low PA was reported in 31% of the participants. D, EE, and low PA were associated significantly with a history of COVID-19 infection and COVID-19 postings	EE was higher in residents, in those who worked in COVID-19 units and were infected with COVID-19, and in those who were unmarried. EE and D were higher in those with lower mental well-being scores, with PA directly proportional to mental well-being. Mental well-being was associated significantly with COVID-19 infection and PA. D was reported more frequently in residents, in those who worked in COVID-19 units and in those infected with COVID-19, regardless of gender or relationship status. PA was observed to be lower in both interns and residents, in those posted in COVID-19 units, in those infected with COVID-19, and in those who were unmarried.	MBI-HSS
Baptista et al, 2021	Portugal	Cross-sectional study	214 physicians	3 dimensions: 65.9% for personal burnout 68.7% for work-related burnout 54.7% for patient-related burnout	A strong association was found between gender, years of professional experience, depression and anxiety, and burnout levels. Participants reported levels of depression (67.3%), anxiety (69.6%), and stress (61.7%). Being a female was significantly associated with higher odds of patient-related burnout. Less experience was also significantly associated with higher odds of patient-related burnout. A reduction in the monthly income was significantly and inversely correlated with patient-related burnout. High levels of depression were significantly associated with higher levels of all 3 burnout dimensions. Higher levels of anxiety were also significantly associated with higher levels of personal burnout.	Copenhagen Burnout Inventory (CBI)
Vila et al, 2022	Argentina	Prospective study	39 cardiologists	Third year residents were the group with the highest incidence of burnout (66.6%), followed by fourth year residents of whom (50%) met the same criteria, and second year residents (40%), affected by burnout syndrome.	There was no identified significant difference in the prevalence of burnout syndrome between both groups according to sex and year of residence.	MBI
Buran & Altin, 2021	Turkey	Cross-sectional study	67 physicians	The EE score was significantly higher in physicians worrying too much about malpractice. PA was significantly higher in physicians feeling the need for training in the medico-legal field. EE score was lower in subspecialist attending physicians, compared to general attending physicians	The EE score was significantly higher in physicians exposed to violence. The notable significance of burnout was related to physicians' anxiety on medico-legal issues and exposure to violence.	MBI

Civantos et al, 2020	Brazil	Cross-sectional study	349 physicians	21.8% of the participants	Anxiety 47.9%, Distress, 60.2%, Depressive symptoms 10.6%. Female physicians were more likely to report burnout	Mini-Z burnout assessment
Civantos et al, 2020	U.S.A.	Cross-sectional study	163 physicians	14.7% burnout	Anxiety, distress and depression were reported in (45.5%), (26.3%), (21.8%), and (16%) physicians, respectively. Attending had decreased burnout relative to residents. Females had increased levels of burnout and distress. Physicians in states with greater than 20 000 positive cases had increased distress.	Mini-Z burnout assessment
Chalhub et al, 2021	Brazil	Cross-sectional study	223 frontline physicians	All physicians on the frontline experienced at least moderate symptoms of burnout.	Physicians with anxiety showed more EE, depersonalization, less PA with anxiety had higher scores in EE, D domains and lower scores in PA.	MBI
Cravero et al, 2021	21 countries	Cross-sectional study	1420 trainees	66% of the trainees who cared for patients with COVID-19 reported burnout, for both EE and D.	Compared with trainees who did not take care of patients with COVID-19, those who took care of 1–30 patients, 31–60 patients and > 60 patients were increasingly more likely to report burnout.	Two validated single-item measures of EE and D
de Wit et al, 2020	Canada	Mixed methods study	468 emergency physicians(EM)	Burnout levels did not significantly change over time	Being tested for COVID-19 in the previous week and the number of shifts worked were associated with high EE. Having been tested for COVID-19 was also associated with high D.	MBI
Dinibutun, 2020	Turkey	Cross-sectional study	200 physicians	EE level was medium. The levels of D and PA were low. Level of total burnout was low.	Burnout levels of males and females and married and single physicians were similar. The EE level of 18-23-year-old physicians was lower than the rest of the physicians. Physicians' level of satisfaction with their income is not effective on burnout. The burnout level of physicians who did not choose their profession willingly was determined to be higher. The burnout level of physicians who were actively involved in the fight against COVID-19 was lower.	MBI
Doherty et al, 2021	Ireland	Cross-sectional study	144 seniors and specialists	77% screened positive for burnout	64% reported that Covid-19 has had an adverse effect on their mental health. 47% of respondents screened positive using cynicism or detachment. 50.9% reported regular feelings of reduced professional ability or accomplishment. 68% reported feeling physically exhausted regularly.	2-item Maslach Burnout Scale (MBS-2)
Faria et al, 2021	Paraiba Brazil	Cross-sectional study	126 physicians	EE – for frontline doctors (high for 50% of the participants, moderate for 39% and low for 11%); for off-frontline doctors (high for 18.2% of the participants, moderate for 68, 2% and low for 13.6 %).<br>D – for frontline physicians (high for 37.8% of the participants, moderate for 54.9% and low for 7.3%); for off-	Burnout was associated with younger age (24-33 years), not having children, working on the front line, working in the COVID-19 ICU, being on duty, and having contracted COVID-19.	MBI-HSS

				frontline doctors (high for 2.3% of the participants, moderate for 79.5%; for 18.2 % was low).<br>PA – for frontline physicians (high for 64.6%, moderate for 32.9% and low for 2.4 %.) For off-frontline doctors (47.7% was high, as well as for moderate level and low for 4.5%)		
Guercovich et al, 2021	Argentina	Cross-sectional study	188 oncologists	25% of the subjects had high levels of D, 39.9% reported high levels of EE, and 53.7% reported low levels of PA.	25% of subjects reported high levels of D, 39.9% reported high levels of EE, and 53.7% reported low levels of PA.	MBI – Spanish version
Jha et al, 2020	U.S.A.	Observational, prospective non-comparative study design	100 physicians	(52%) of the physicians felt burnt out. Top three reasons contributed for the state of exhaustion and were attributed to financial stress (23%), administrative burden (11%), and uncertainty (12%)	67% of the physicians responded that in-house billing was responsible for their increased level of burnout. 73% of the physicians responded that electronic medical records (EMRs) were one of the causes. Overall, 78% were very concerned.	32-question survey
Kannampallil et al, 2020	U.S.A.	Cross-sectional study	393 physicians - trainees	The exposed group had a higher prevalence of burnout (46.3% vs. 33.7%)	The exposed group to patients who were being tested for COVID-19 experienced moderate to extremely high perceived stress regarding childcare (61.7% vs. 39.2%), reported considerably lower work-family balance (sometimes or often, 68.2% vs. 55.4%) and more difficulty in taking time off for attending to personal or family matters (somewhat or very hard, 74.1% vs. 47.7%), had a higher prevalence of stress (29.4% vs. 18.9%), and burnout (46.3% vs. 33.7%); higher prevalence of anxiety (21.6% vs. 14.9%). The exposed and non-exposed group had similar prevalence of depression (28% vs. 26.3%). Both groups had low professional fulfilment from their current clinical work activities (25.2% vs. 25.9%). Usage of wellness resources by trainees was low (5 %).	Stanford Professional Fulfilment Index (PFI)
Kase et al, 2022	U.S.A.	Cross-sectional study	499 paediatricians	Higher BO scores were significantly associated with higher compassion fatigue score, emotional depletion.	Besides compasion fatigue, other significant predictors of burnout were the feeling of „Self care is not a priority for me" and emotional depletation. At the oposite pole, "My institution has valued my contribution to the Covid-19 crisis" was associated with lower burnout scores"	CFST
Khalafallah et al, 2020	U.S.A.	Cross-sectional study	407 neurosurgeons	The rate of burnout among respondents was 20.4 %	Burnout was associated with working in a hostile or difficult environment (COVID-19), not having children, being in practice for 5-15 years, spending increased time conducting non-neurosurgical medical care because of COVID-19, feeling uncertain about future earnings due to COVID-19, choosing not to pursue or feeling uncertain about pursuing neurosurgery again.	The abbreviated Maslach Burnout Inventory (aMBI)

Khan et al, 2021	Canada	Cross-sectional study	302 physicians	The prevalence of burnout was 68% (EE - 63%, D - 39% and PA - 22%)	Women were more likely to report EE and feel low PA. Visible ethnic minority physicians were more likely to report feeling lower personal accomplishment than white physicians. Physicians who reported that COVID-19 affected their burnout were more likely to report any burnout and consideration of quitting or quitting.	MBI
Lazarides et al, 2021	U.S.A.	Cross-sectional study	63 orthopaedic surgeons	Personal fulfilment (42.8%) Burnout - (15.9%)	There was no relationship between burnout or professional fulfilment and gender.	MBI
Lou et al, 2021	U.S.A.	Cross-sectional study	301 trainees	52% of the trainees who reported being burned out were concerned about missed educational opportunities, as compared to 42% in the non-burned-out group. Professional fulfilment was not significantly associated with concern for missed educational opportunities.	Trainees assigned to education at home had 2.85 greater odds of being concerned over missed educational opportunities as compared to trainees performing clinical work. Trainees performing research were not similarly affected. Trainees in pathology or radiology had greater odds of concern for missed educational opportunities as compared to trainees in medicine.	Stanford Professional Fulfilment Index (PFI)
Macía-Rodríguez et al, 2021	Spain	Cross-sectional study	1015 internists	58.3% presented high EE; 61.5% had a high level of D; 67.6% reported low PA; 40.1% presented with the 3 criteria described, and therefore burn-out syndrome.	Burnout syndrome was independently related to the management of patients with SARS-CoV-2, the lack of availability of personal protective equipment, increased responsibility, not having received financial compensation for overtime work, not having rested after 24-hour shifts, not having had holidays in the previous 6 months, consumption of sleeping pills, and higher alcohol intake.	MBI
Mendonça et al, 2021	Brazil	Cross-sectional study	3071 medical residents	Burnout (55.2%) among fourth-year residents and (55.1%) among second-year residents.	Depressive symptoms were the most common among second-year residents (70.5%), followed by anxiety symptoms (56.0%).	OLBI

Nguyen et al, 2021	U.S.A.	Cross-sectional study	166 emergency physicians (EM)	Burnout was reported by 74.7% of the respondents	Physicians who felt there was inadequate institutional support were dissatisfied with patient care resources. Physicians expressing job dissatisfaction were more likely to report feelings of burnout. There was reported an association between burnout and age (being younger), as well as gender. The highest proportions of reported burnout were those between 41 and 50 years of age; those in the oldest age group, 71–75, reported burnout in the lowest proportions. Of the physicians reporting burnout, 70.2% were white, 67.7% were male, 34.7% were employed at community teaching hospitals, 46.8% have been in practice for >20 years, and 37.1% were in the Southern US. The most common feelings associated with burnout were increased work-related emotional strain and anxiety, followed by isolation from family and friends, and then by increased workload.	MBI-HSS
Ofei-Dodoo et al, 2021	U.S.A.	Cross-sectional study	113 family physicians	50.4% of the respondents reported manifestations of burnout	The physicians who personally treated any presumptive or confirmed COVID-19 patients were more likely to report at least one manifestation of burnout, experience EE, and feel a higher level of personal stress.	2 single-item measures of EE and D adapted from the full (MBI)
Park et al, 2020	Republic of Korea	Cross-sectional study	115 physicians	Burnout reported 90.4%	Similar burnout scores between the sexes. Female physicians tended to report depression, anxiety, and stress more frequently than males and a higher proportion of were found to endorse EE.	MBI-HSS
Rodriguez et al, 2021	U.S.A.	Cross-sectional study	426 EM physicians	Reported higher levels of EE/burnout since the pandemic started	Most physicians (90.8%) reported changing their behaviour toward family and friends, especially by decreasing signs of affection (76.8%). The most cited measures referred to alleviate stress/anxiety were increasing.	32 Likert-type scale
Seda-Gombau et al, 2021	Spain	Multicentre longitudinal descriptive study	40 physicians responded to the three surveys	In January 2019, 10% of primary care physicians scored high on all burnout domains. In October 2020, this percentage increased to 50%. Unprecedented worsening due to the pandemic: EE, which already affected 55% of primary care physicians, jumped to 77%	Being older increases the chances of having burnout. Significant variable was sleep; specifically, individuals not sleeping well scored on average. The lack of sleep was significant for EE.	MBI-HSS
Tuna & Özdin, 2021	Turkey	Descriptive study	406 physicians	Physicians working in COVID units had higher levels of D. PA was low among research assistants and in participants working in university research hospitals.	Female gender, lack of COVID training, difficulty obtaining protective equipment, working in a COVID unit, and current psychiatric disease predicted desensitization.	MBI

Yao et al, 2021	U.S.A.	Cross-sectional study	870 physicians - breast oncologists	Amongst the COVID-19 related burnout emotions, EE was reported with the highest frequency (33.6%), "anxious" was the second most common and "burnout" was the third most common. Physician anxiety, sleep disturbance and COVID-19 related burnout were significantly associated with demographic factors, including COVID-19 testing factors.	Mean anxiety and COVID-19 burnout scores were significantly higher among physicians whose patients experienced either delays in surgery, adjuvant chemotherapy, radiation, breast imaging or specialty consultation. Anxiety and COVID-19 related burnout were significantly associated with physician age, gender and years in practice, and sleep disturbance was significantly associated with physician age. There were no significant differences in physician anxiety, sleep disturbance and COVID-19 related burnout between different facility locations, practice settings or physician specialty type. Physicians who took care of COVID-19 positive patients reported significantly higher anxiety, sleep disturbance and COVID-19 related burnout than physicians who did not take care of COVID-19 positive patients.	6 COVID-19 burnout emotions
